# Inhibitory Effects of Solvent-Partitioned Fractions of Two Nigerian Herbs (*Spondias mombin* Linn. and *Mangifera indica* L.) on α-Amylase and α-Glucosidase

**DOI:** 10.3390/antiox7060073

**Published:** 2018-05-26

**Authors:** Oluwafemi Adeleke Ojo, Adeola Agnes Afon, Adebola Busola Ojo, Basiru Olaitan Ajiboye, Babatunji Emmanuel Oyinloye, Abidemi Paul Kappo

**Affiliations:** 1 Phytomedicine, Biomedical Toxicology and Diabetes Research Laboratories, Department of Biochemistry, Afe Babalola University, Ado-Ekiti 360001, Nigeria; adeolabunmi24@gmail.com (A.A.A.); ajiboyebo@abuad.edu.ng (B.O.A.); babatunjioe@abuad.edu.ng (B.E.O.); 2 Department of Biochemistry, University of Ilorin, Ilorin 240222, Nigeria; 3 Department of Medical Biochemistry, Afe Babalola University, Ado-Ekiti 360001, Nigeria; ojoab@abuad.edu.ng; 4 Biotechnology and Structural Biology (BSB) Group, Department of Biochemistry and Microbiology, University of Zululand, KwaDlangezwa 3886, South Africa; kappoA@unizulu.ac.za

**Keywords:** *Spondias mombin*, *Mangifera indica*, α-amylase, α-glucosidase, antioxidant activity

## Abstract

Therapies directed towards controlling hyperglycemia, the hallmark of type-2 diabetes mellitus, go a long way in managing diabetes and its related complications. Reducing glucose level through the inhibition of the relevant carbohydrate hydrolyzing enzymes is one among many routes in the management of diabetes. This study investigates the *in vitro* enzyme inhibitory and antioxidant properties of solvent-partitioned fractions of *Spondias mombin* and *Mangifera indica* leaves; which are used extensively in the treatment of diabetic patients locally. The leaves of *S. mombin* and *M. indica* were extracted with methanol and fractionated to obtain *n*-hexane (HF), ethyl acetate (EAF), *n*-butanol (BF), and aqueous (AF) fractions successively. The α-amylase and α-glucosidase inhibitory activities of fractions of *S. mombin* and *M. indica* leaves were investigated while the antioxidant activity of each fraction was analyzed using iron chelating and ABTS (2,2’-azino-bis(3-ethylbenzothiazoline)-6-sulphonic acid) radical scavenging assay. Our findings indicated that the ethyl acetate fraction of *M. indica* leaves contained a considerably higher (*p* < 0.05) amount of total phenolic, flavonoids, metal ion, and ABTS radical scavenging activity than the ethyl acetate fractions of *S. mombin*. Furthermore, the ethyl acetate fraction of *M. indica* had a considerably higher (*p* < 0.05) inhibitory effect on α-glucosidase (IC_50_ = 25.11 ± 0.01 μg mL^−1^), and α-amylase (IC_50_ = 24.04 ± 0.12 μg mL^−1^) activities than the *S. mombin* fraction. Hence, the inhibitory activities of *S. mombin* and *M. indica* leaves suggest that they are a potential source of orally active antidiabetic agents and could be employed to formulate new plant-based pharmaceutical and nutraceutical drugs to improve human health.

## 1. Introduction

Diabetes mellitus (DM) is a major public health problem. The projected prevalence among adults in 2015 was 8.8%, affecting about 415 million adults. The prevalence of diabetes has been predicted to increase to about 10.4% by 2040 [1]. The recent exponential increase in the prevalence of this chronic disease requires a multiple therapeutic approach in the search of a real solution for diabetes and this includes the development of other alternative or complementary medications. Evidence from traditional prescription and scientific investigation reveals optimum therapeutic efficacy of medicinal plants with a good margin of safety. Since medicinal plants form a major part of human food, it is worthwhile to evaluate their inhibitory activity against hyperglycemia [2,3].

Hyperglycemia is considered the major basis for many problems in the diabetic state. Meanwhile, carbohydrates are the main sources of blood glucose and inhibition of relevant enzymes such as α-amylase and α-glucosidase associated with non-insulin dependent diabetes mellitus is vital in preventing sudden increase in blood glucose. Hence, the inhibition of theses enzymes is the reason why the digestion process of carbohydrates can be retarded and the absorption rate of glucose from the gut decreased to result in an extreme low level of blood glucose. As it was previously mentioned, the ability to maintain low blood glucose level is the hallmark of the treatment of diabetes. Although, this may be accomplished via the use of a standard therapy regimen such as biguanides and insulin secretagogues, the inhibition of α-amylase and α-glucosidase is another key therapeutic approach to be explored in order to improve glycemic control [4,5]. Additional searches for plant-based antidiabetic components would be valuable as revealed by their prominent role in some of the presently accessible orthodox drugs [6]. 

*Spondias mombin* L. (Anarcadiaceae) is called “Hog plum”, “*Iyeye”* and “*Olosan*” (Yoruba) [7]. It is a tree with giant panicles of little white flowers, frequent in farmland and villages, particularly within the forest region in addition to the savannah. In traditional practice, *S. mombin* is employed in curing duodenal disorders, gonorrhoea, diabetes, psychiatric disorders, and for the removal of the placenta in childbirth. In addition, it is used as an antidiarrheal agent [8], and as an antimicrobial agent as well as for the healing of wounds [9,10,11]. Iweala and Oludare, also reported the hypoglycemic effects, as well as the biochemical and histological changes of the ethanolic extract of *Spondias mombin* in alloxan-induced diabetic rats [12]. Pelandjuaic acid, ellagitannins, caffeoyl esters, and anacardic acid are reported to be present in *S. mombim* [13,14,15]. 

*Mangifera indica* L. (Anacardiaceae) called “Mango”, “*Mangoro*” (Yoruba) is a perennial tree prevalent in rural and semi-urban parts of Nigeria. It is one of the vital tropical plants marketed in the world [16]. It is grown largely in several parts of Africa, particularly in the western parts of Nigeria, where it is valued for its edible fruits. There are numerous conventional uses for the bark, roots, and leaves of *M. indica* throughout the globe. *M. indica* is used therapeutically to cure several ailments such as asthma, cough, diarrhea, dysentery, leucorrhoea, jaundice, pain, and malaria. Phytochemical studies from different parts of *M. indica* have revealed the presence of phenolic constituents, triterpenes, flavonoids, phytosterol, and polyphenols [17]. *M. indica* is believed to possess several therapeutic uses including analgesic, anti-inflammatory, antimicrobial as well as immune-stimulant, antioxidant and antilipidemic applications [18,19]. Ethanol extract of *M. indica* peel has been investigated and reported to inhibit α-amylase and α-glucosidase activities, and to ameliorate diabetes related biochemical parameters in streptozotocin (STZ)-induced diabetic rats [20]. Previous studies have reported extraction of different chemical compounds such as phenolic acid 6-alkenyl-salicylic acid, anarcardic acid, chlorogenic acid, ellagic acid, betulin, coumaroyl, quercetin, and gallic acid from *S. mombin*, which exhibited different biological activities including antidiabetic, anti-inflammatory and anti-oxidant effects [15,21,22,23,24]. On the other hand, studies have reported that bioactive compounds identified from *M. indica* leaves include quercetin and chlorogenic acid which possess anti-oxidant, anti-inflammatory, and antidiabetic activities [25]. The current study aimed to investigate the *in vitro* enzyme inhibitory activities and antioxidant properties of the *S. mombin* and *M. indica* leaf solvent-partitioned fractions as potential therapeutic sources, which may be helpful in attaining the normoglycemic state in the diabetic condition.

## 2. Materials and Methods

### 2.1. Chemicals

All chemical agents and standards were of analytical grade reagents unless otherwise stated. Folin–Ciocalteu’s reagent and methanol were purchased from Merck (Darmstadt, Germany). ABTS radical cation (2,2’-azino-bis(3-ethylbenzothiazoline)-6-sulphonic acid), DTNB (5,5-dithio-bis (2-nitrobenzoic) acid), acarbose, α-amylase, and α-glucosidase were purchased from Sigma-Aldrich (Steinheim, Germany). 

### 2.2. Plant Material and Extraction Procedure

Fresh leaves of *S. mombin* and *M. indica* were obtained from Ibadan in Nigeria in September 2017. Fresh leaves of *S. mombin* and *M. indica* were identified and documented by Mr. Odewo from Forestry Research Institute of Nigeria (FRIN) with Forest Herbarium Ibadan: FHI 111312 and FHI 111313, respectively as the herbarium number deposited. The fresh leaves were air-dried at normal room temperature and humidity for three weeks and ground to powder using a mechanical blender. To obtain methanol extracts, 100 g of air-dried leaves were soaked with 800 mL of methanol and 200 mL of water for 48 h [26]. The methanol extract obtained was concentrated using a rotary evaporator and stored until use. 

### 2.3. Solvent-Partitioned Fractionation of Crude Methanol Extracts

Methanol leaves extract of *S. mombin* (9 g) and *M. indica* (15.5 g) was solubilized in 200 mL of distilled water and sequentially extracted with solvents of increasing polarity (hexane, ethyl acetate, and *n*-butanol). Methanol extract was partitioned between *n*-hexane (2 × 200 mL) and water to obtain an *n*-hexane fraction (HF) and an aqueous portion. The aqueous portion obtained was further partitioned by ethyl acetate (2 × 200 mL) to obtain an ethyl acetate fraction (EAF) and an aqueous portion. The aqueous portion obtained was further partitioned by *n*-butanol (2 × 200 mL) to obtain an *n*-butanol fraction (BF) and a residual aqueous fraction (AF). 

### 2.4. α-Amylase Inhibitory Activity of Fractions of S. mombin and M. indica Leaves

Alpha-amylase activity was assessed concurring to the protocol described by [27] with slight modification by [28]. A volume of 300 μL of *S. mombin* and *M. indica* leaf fractions (HF, EAF, BF, AF) at different concentrations (10–150 μg mL^−1^) was incubated with 500 μL of porcine pancreatic amylase (2 U mL^−1^) in 100 mmol L^−1^ phosphate buffer (pH 6.8) at 37 °C for 20 min. Three hundred μL of 1% starch dissolved in 100 mmol L^−1^ phosphate buffer (pH 6.8) was then added to the mixture and incubated at 37 °C for 1 h. One mL of dinitrosalicylic acid (DNS) color was then added to the solution and boiled for 10 min. The absorbance of the ensuing mixture was read at 540 nm and the enzyme inhibitory activity was calculated as percentage of control sample without inhibitors. Acarbose was used as standard.

$$\alpha -\mathrm{amylase}\;\mathrm{inhibition}\;\left(\%\right)=\;\frac{A_{540\mathrm{control}}-A_{540\mathrm{sample}}}{A_{540\mathrm{control}}}\;\times 100$$

A_540control_: Absorbance of control at 540 nm; A_540sample_: Absorbance of sample at 540 nm

### 2.5. α-Glucosidase Inhibitory Activity of Fractions of S. mombin and M. indica Leaves

Alpha-glucosidase inhibitory activity was determined in line with the protocol by [29], with small alterations by [30]. Briefly, 300 μL of *S. mombin* and *M. indica* leaf fractions (HF, EAF, BF, AF), at varying concentrations (10–150 μg mL^−1^), was mixed with 500 μL of 1.0 U mL^−1^ α-glucosidase solution in 100 mmol L^−1^ phosphate buffer (pH 6.8) at 37 °C for 15 min. Afterwards, 300 μL of p-nitrophenyl-α-D-glucopyranoside (pNPG) solution (5 mmol L^−1^) in 100 mmol L^−1^ phosphate buffer (pH 6.8) was added and then the solution was further mixed at 37 °C for 20 min. Absorbance of the free *p*-nitrophenol was read at 405 nm and then the inhibitory activity was expressed as percentage of a the control sample. Acarbose was used as standard.

$$\alpha -\mathrm{glucosidase}\;\mathrm{inhibition}\;\left(\%\right)=\;\frac{A_{405\mathrm{control}}-A_{405\mathrm{sample}}}{A_{405\mathrm{control}}}\;\times 100$$

A_405control_: Absorbance of control at 405 nm; A_405sample_: Absorbance of sample at 405 nm

### 2.6. Estimation of Total Phenol Content

*S. mombin* and *M. indica* phenol content of the leaf fractions (HF, EAF, BF, AF) was estimated as described by [31]. In short, 200 μL fractions (HF, EAF, BF, AF) dispersed in 10% dimethylsulfoxide (DMSO) (240 μg mL^−1^) was incubated with 1.0 mL of Folin Ciocalteau (diluted 10 times) and 800 μL of 0.7 mol L^−1^ Na_2_CO_3_ for 30 min. Absorbance was read at 765 nm and all readings were in triplicate with results expressed as mg gallic acid equivalents (GAE)/100 g dry fractions.

### 2.7. Estimation of Flavonoid Content

*S. mombin* and *M. indica* leaf fractions (HF, EAF, BF, AF) were estimated for flavonoid content using the procedure described by [32]. Briefly, 0.5 mL of suitably diluted sample was mixed with 0.5 mL methanol, 50 μL 10% AlCl_3_, 50 μL 1 M potassium acetate, and 1.4 mL water, and incubated at room temperature for 30 min. Absorbance of the solution was read at 415 nm. All experiments were in triplicate. 

### 2.8. Evaluation of Antioxidant Activities of Fractions of S. mombin and M. indica Leaves

#### 2.8.1. Iron (Fe^2+^) Chelation

The metal chelating property of *S. mombin* and *M. indica* leaf fractions (HF, EAF, BF, AF) was determined by employing an altered procedure of [33]. Freshly prepared 500 μmol L^−1^ FeSO_4_ (150 μL) was mixed to the solution comprising 168 μL of 0.1 mol L^−1^ Tris-HCl (pH 7.4), 218 μL saline, the aqueous extract (10–150 μL), and the fractions. The solution was incubated for 5 min, with addition of 13 μL of 0.25% (*w/v*) of 1,10-phenanthroline. Ethylenediaminetetraacetic acid (EDTA) was used as standard. Absorbance was read at 510 nm. 

#### 2.8.2. Estimation of 2,2-Azino-bis3-ethylbenthiazoline-6sulphonic acid (ABTS) Radical Scavenging Ability

*S. mombin* and *M. indica* leaf fractions (HF, EAF, BF, AF) were assessed primarily based on the ability to scavenge ABTS using the protocol delineated by [34]. The ABTS was produced by reacting 7 mM ABTS aqueous solution with K_2_S_2_O_8_ (2.45 mM) in the dark for 16 h and altering the absorbance at 734 nm. Afterward, 200 μL of suitable dilution of extracts and fractions was added to 2.0 mL ABTS solution. Vitamin C was used as standard. Absorbances were read at 734 nm after 15 min. 

### 2.9. Data Analysis

Results were expressed as the mean ± standard error of mean (SEM) of triplicates [35] from independent samples. Level of significance was set to *p* < 0.05. These analyses were presented using one-way analysis of variance (ANOVA) using SPSS version 21.0 (IBM Corporation, NY, USA).

## 3. Results

### 3.1. Inhibitory Effect of Various Fractions of S. mombin and M. indica Leaves against α-Amylase

**Figure 1:**
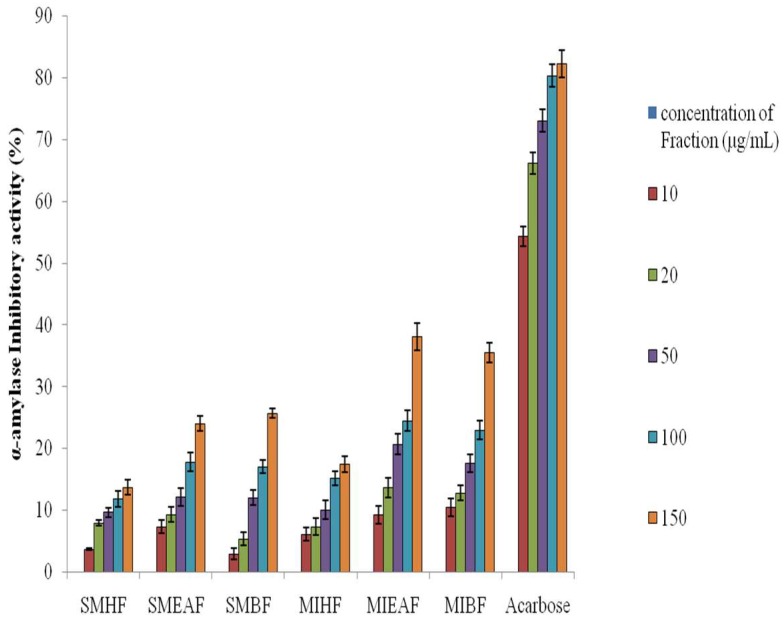
Alpha-amylase inhibitory activity of fractions of *S. mombin* and *M. indica* leaves. Legends: SMHF: *S. mombin n*-hexane fraction; SMEAF: *S. mombin* ethyl acetate fraction; SMBF: *S. mombin n*-butanol fraction; MIHF: *M. indica n*-hexane fraction; MIEAF: *M. indica* ethyl acetate fraction; MIBF: *M. indica n*-butanol fraction.

Figure 1 shows the inhibition percentage of α-amylase by various fractions of crude methanol extract of *S. mombin*, *M. indica* leaves and standard drug acarbose. The *M. indica* fractions had appreciable *in vitro* inhibitory activity against α-amylase in a fashion, with the ethyl acetate fraction (IC_50_ = 24.04 ± 0.12 μg mL^−1^) showing a considerably better (*p* < 0.05) α-amylase inhibitory activity than *S. mombim* leaf (IC_50_ = 28.12 ± 0.48 μg mL^−1^) fractions. However, acarbose had the highest activity against α-amylase as shown by the IC_50_ (22.08 ± 0.03 μg mL^−1^).

### 3.2. Inhibitory Effect of Various Fractions of S. mombin and M. indica Leaves against α-Glucosidase

**Figure 2:**
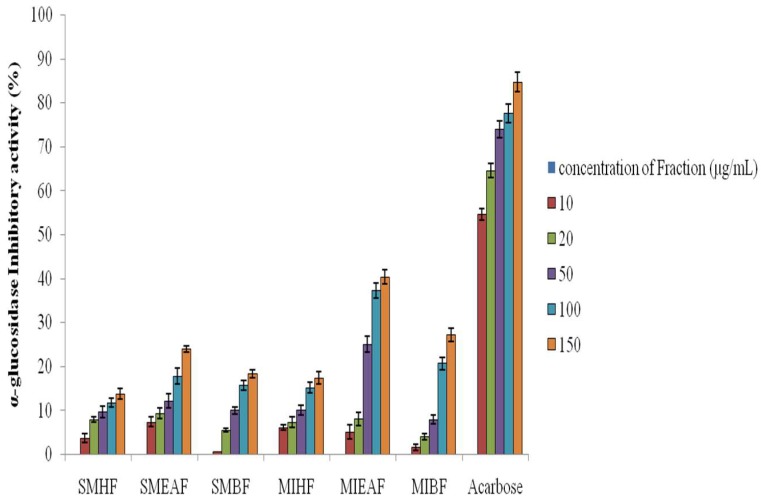
Alpha-glucosidase inhibitory activities of fractions of *S. mombin* and *M. indica* leaves**.** Legends: SMHF: *S. mombin n*-hexane fraction; SMEAF: *S. mombin* ethyl acetate fraction; SMBF: *S. mombin n*-butanol fraction; MIHF: *M. indica n*-hexane fraction; MIEAF: *M. indica* ethyl acetate fraction; MIBF: *M. indica n*-butanol fraction.

Figure 2 shows the percentage inhibition of α-glucosidase by various fractions of crude methanol extract of *S. mombin* and *M. indica* leaves. The *M. indica* fractions inhibited α-glucosidase activities in vitro. The ethyl acetate fraction displays a better inhibition of α-glucosidase activity compared to other fractions. Notably, the inhibitory activity of the ethyl acetate fraction of *M. indica* (IC_50_ = 25.11 ± 0.01 μg mL^−1^) was considerably higher (*p* < 0.05) than *S. mombim* (IC_50_ = 12.05 ± 0.02 μg mL^−1^) fraction as indicated by their IC_50_ values. However, acarbose had a better inhibitory activity against α-glucosidase than *S. mombin* and *M. indica* leaves.

### 3.3. Total Phenolics and Total Flavonoids Content of Fractions of S. mombin and M. indica Leaves

Table 1 reveals the total phenolics, total flavonoids, by various fractions of crude aqueous extract of *S. mombin* and *M. indica* leaves. The ethyl acetate fraction of *M. indica* leaves (193.49 ± 18.64 mg GAE/100 g) had considerably (*p* < 0.05) higher phenol content than *S. mombin* ethyl acetate fraction (33.44 ± 1.57 mg GAE/100 g). Also, the ethyl acetate fraction of *M. indica* (52.35 ± 1.23 mg AAE/100 g) had appreciably (*p* < 0.05) higher flavonoids (Table 1) than *S. mombin* ethyl acetate fraction (19.86 ± 2.89 mg QUE (Quercetin equivalents)/100 g).

### 3.4. Metal Ion Chelating Ability of Fractions of S. mombin and M. indica Leaves

**Figure 3:**
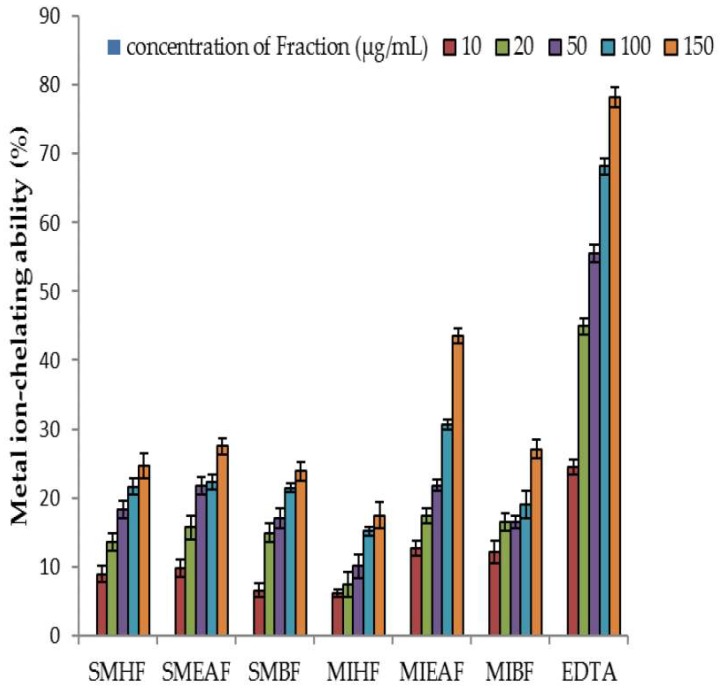
Metal ion chelating property of fractions of *S. mombin* and *M. indica* leaves. Legends: SMHF: *S. mombin n*-hexane fraction; SMEAF: *S. mombin* ethyl acetate fraction; SMBF: *S. mombin n*-butanol fraction; MIHF: *M. indica n*-hexane fraction; MIEAF: *M. indica* ethyl acetate fraction; MIBF: *M. indica n*-butanol fraction; EDTA: ethylenediaminetetraacetic acid.

The metal ion chelating property of various fractions of *S. mombin* and *M. indica* leaves is displayed in Figure 3. This demonstrated that the ethyl acetate fraction of *S. mombin* (IC_50_ = 21.76 ± 0.02 μg mL^−1^) had a considerably (*p* < 0.05) higher metal chelating property than *M. indica* (IC_50_ = 21.82 ± 0.05 μg mL^−1^), ethyl acetate fractions. However, EDTA had a metal-ion chelating ability better than the fractions.

### 3.5. Antioxidant Capacity of Fractions of S. mombin and M. indica Leaves

**Figure 4:**
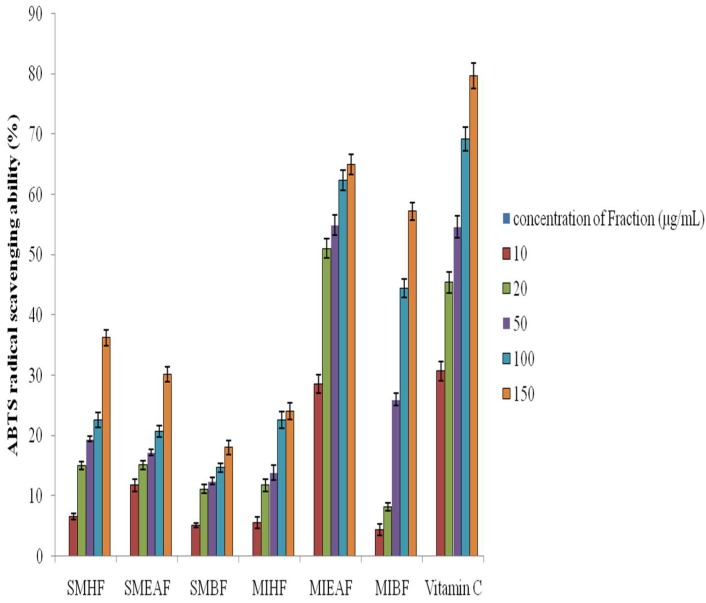
ABTS (2,2’-azino-bis(3-ethylbenzothiazoline)-6-sulphonic acid) radical scavenging ability of fractions of *S. mombin* and *M. indica* leaves. Legends: SMHF: *S. mombin n*-hexane fraction; SMEAF: *S. mombin* ethyl acetate fraction; SMBF: *S. mombin n*-butanol fraction; MIHF: *M. indica n*-hexane fraction; MIEAF: *M. indica* ethyl acetate fraction; MIBF: *M. indica n*-butanol fraction.

The free radical scavenging ability of the various fractions of *S. mombin* and *M. indica* leaves was consequently evaluated using the abstemiously steady ABTS radical and is displayed in Figure 4. Results showed that the ethyl acetate fraction of *M. indica* (IC_50_ = 54.88 ± 0.01 μg mL^−1^) quenched ABTS radical (20–100 μg mL^−1^) better than *S. mombin* ethyl acetate leaves (IC_50_ = 17.15 ± 0.02 μg mL^−1^), fractions as indicated by their IC_50_ values. However, vitamin C scavenged ABTS radical better than the fractions.

## 4. Discussion

Although several scientific studies have reported the antioxidant and antidiabetic activities of numerous medicinal plants including *M. indica and S. mombin* [36,37,38,39,40,41], to the best of our knowledge, this is the first report that directly compares the inhibitory effects of solvent-partitioned fractions of *M. indica and S. mombin* on α-amylase and α-glucosidase. There are several therapeutic approaches for managing diabetes mellitus; one way to achieve controlled blood glucose levels is to delay glucose absorption via inhibition of relevant carbohydrate hydrolyzing enzymes, such as α-amylase and α-glucosidase, found in the small intestine. The present study showed that *S. mombin* and *M. indica* leaves (HF, EAF, BF, AF) fractions inhibit α-amylase and α-glucosidase activities. The inhibition of carbohydrate metabolizing enzymes like α-amylase and α-glucosidase retards the absorption and digestion of starch and later suppresses postprandial symptom. The inhibitory properties of *S. mombin* and *M. indica* leaf fractions may suggest its usefulness as an oral antidiabetic drug for the management of high blood sugar in patients with these syndromes. Inhibitions of these enzymes interrupt macromolecule digestion and overall extend the breakdown time inflicting a reduction in the degree of glucose ingestion and thus plummeting postprandial blood sugar [30]. Better medical output may be derived from α-amylase and α-glucosidase inhibitors with mild inhibitory activity against α-amylase and strong inhibitory activity against α-glucosidase [42]. The inhibition of α-glucosidase, together with α-amylase by ethyl acetate fractions of *M. indica* and *S. mombin*, is considered to be an effective strategy for the control of diabetes by diminishing the absorption of glucose [42,43]. Remarkably, in this study, the ethyl acetate fractions of *M. indica* and *S. mombin* validated these properties and hence could be considered for therapeutic approach to retard postprandial hyperglycemia.

Recently, phenolic compounds have attracted great interest for their potential use in the development of new nutraceuticals or pharmaceuticals products due to their remarkable anti-oxidant, anti-inflammatory or antibacterial activities. Although, the protective effects of polyphenols could be in a concentration-dependent manner, recently there has been accumulating evidence in support of the hypothesis that a high-concentration of polyphenols can mechanistically cause adverse effects through pro-oxidative action and negatively affect cell growth, causing toxicity [43]. Several of the present antioxidants show mutagenic and genotoxic responses in cells reflecting their oxidant activity [44,45]. Flavonoids are major classes of phenolics and many studies have documented their biological and pharmacological activities [46,47]. The phenolic contents of *M. indica* and *S. mombin* fractions were determined respectively and the ethyl acetate fraction of *M. indica* leaves had higher total phenolic and flavonoid content than *S. mombin* fractions.

Metal ion chelating ability is important since it reduces the concentration of transition metals [48]. By chelating Fe^2+^, the generation of hydroxyl radicals in the Fenton reaction may be attenuated and thus prevent damage to biomolecules. Accumulation of iron has been reported to cause an elevation in the generation of free radicals and development of oxidative stress [49,50].

Rice-Evans [51] reported that compounds with phenolic content could play an important role in eliminating radicals. The ABTS∙ scavenging property of the leaf might be due to the donating ability of the phenolics present in the fractions [52,53,54]. The antioxidant capacity of the leaves can be linked to their bioactive compounds, mainly antioxidant polyphenols, because of their ability to scavenge free radicals [55]. On this note, we suggest that the phenolic acids present in the fractions of *M. indica* and *S. mombin* could contribute to the fraction antioxidant activity. Hence, the results might be explained by the higher total phenolic content found in the fraction of *M. indica* and *S. mombin*. Similar findings were reported by other researchers, who found a strong correlation between radical scavenging ability and total phenolic contents of different samples [56]. However, the ethyl acetate fraction of *M. indica* and *S. mombin* leaf revealed the highest radical reducing ability of all other fractions.

## 5. Conclusions

Conclusively, our results demonstrate that the fractions from *S. mombin* and *M. indica* leaves exert an inhibitory activity against α-amylase and α-glucosidase. This study recommends the use of these plants for further in vivo studies to determine their potential in the management of diabetes. In addition, the data obtained compliments the conventional use of *S. mombin* and *M. indica* in the management of diabetes.

## Figures and Tables

**Table 1:** Total phenolic and total flavonoid content of fractions of *Spondias mombin* and *Mangifera indica* leaves.

Parameters/Fractions	SMHF	SMEAF	SMBF	MIHF	MIEAF	MIBF
Total Phenolic (mg GAE/100 g)	5.23 ± 0.31	33.44 ± 1.57	7.73 ± 1.73	8.10 ± 2.69	193.49 ± 18.64	47.73 ± 2.21
Total Flavonoid (mg QUE/100 g)	3.36 ± 1.41	19.86 ± 2.89	5.75 ± 0.88	4.21 ± 0.85	52.35 ± 1.23	17.01 ± 0.44

Values are given as mean ± standard error of mean (SEM) (*n* = 3). QUE: Quercetin equivalents; GAE: Gallic acid equivalents; SMHF: *S. mombin n*-hexane fraction; SMEAF: *S. mombin* ethyl acetate fraction; SMBF: *S. mombin n*-butanol fraction; MIHF: *M. indica n*-hexane fraction; MIEAF: *M. indica* ethyl acetate fraction; MIBF: *M. indica n*-butanol fraction.
